# Predicting short-term outcomes in brain-injured patients: a comprehensive approach with transcranial Doppler and intracranial compliance assessment

**DOI:** 10.1007/s10877-024-01181-y

**Published:** 2024-06-06

**Authors:** Sérgio Brasil, Danilo Cardim, Juliana Caldas, Chiara Robba, Fabio Silvio Taccone, Marcelo de-Lima-Oliveira, Márcia Harumy Yoshikawa, Luiz Marcelo Sá Malbouisson, Wellingson S. Paiva

**Affiliations:** 1 Division of Neurosurgery, Department of Neurology, School of Medicine, University of São Paulo, Av. Dr. Eneas de Carvalho Aguiar 255, São Paulo, Brazil; 2 Department of Anesthesiology, School of Medicine, University of São Paulo, São Paulo, Brazil; 3 Dipartimento di Scienze Chirurgiche Diagnostiche e Integrate, Università di Genova, Genova, Italy; 4 Anesthesia and Intensive Care, IRCCS Policlinico San Martino, Genova, Italy; 5 Department of Intensive Care, Hôpital Universitaire de Bruxelles (HUB), Université Libre de Bruxelles (ULB), Brussels, Belgium; 6 Escola Bahiana de Medicina e Saúde Pública, Salvador, Bahia Brazil; 7 Instituto D’Or de Pesquisa e Ensino (IDOR), Rio de Janeiro, Brazil; 8 Brain4care, Johns Creek, USA

**Keywords:** Acute brain injury, Cerebral hemodynamics, Intracranial compliance, Intracranial pressure, Transcranial Doppler, Traumatic brain injury

## Abstract

Neurocritical patients frequently exhibit abnormalities in cerebral hemodynamics (CH) and/or intracranial compliance (ICC), all of which significantly impact their clinical outcomes. Transcranial Doppler (TCD) and the cranial micro-deformation sensor (B4C) are valuable techniques for assessing CH and ICC, respectively. However, there is a scarcity of data regarding the predictive value of these techniques in determining patient outcomes. We prospectively included neurocritical patients undergoing intracranial pressure (ICP) monitoring within the first 5 days of hospital admission for TCD and B4C assessments. Comprehensive clinical data were collected alongside parameters obtained from TCD (including the estimated ICP [eICP] and estimated cerebral perfusion pressure [eCPP]) and B4C (measured as the P2/P1 ratio). These parameters were evaluated individually as well as in combination. The short-term outcomes (STO) of interest were the therapy intensity levels (TIL) for ICP management recommended by the Seattle International Brain Injury Consensus Conference, as TIL 0 (STO 1), TIL 1–3 (STO 2) and death (STO 3), at the seventh day after last data collection. The dataset was randomly separated in test and training samples, area under the curve (AUC) was used to represent the noninvasive techniques ability on the STO prediction and association with ICP. A total of 98 patients were included, with 67% having experienced severe traumatic brain injury and 15% subarachnoid hemorrhage, whilst the remaining patients had ischemic or hemorrhagic stroke. ICP, P2/P1, and eCPP demonstrated the highest ability to predict early mortality (*p* = 0.02, *p* = 0.02, and *p* = 0.006, respectively). P2/P1 was the only parameter significant for the prediction of STO 1 (*p* = 0.03). Combining B4C and TCD parameters, the highest AUC was 0.85 to predict death (STO 3), using P2/P1 + eCPP, whereas AUC was 0.72 to identify ICP > 20 mmHg using P2/P1 + eICP. The combined noninvasive neuromonitoring approach using eCPP and P2/P1 ratio demonstrated improved performance in predicting outcomes during the early phase after acute brain injury. The correlation with intracranial hypertension was moderate, by means of eICP and P2/P1 ratio. These results support the need for interpretation of this information in the ICU and warrant further investigations for the definition of therapy strategies using ancillary tests.

**Supplementary Information:**

The online version contains supplementary material available at 10.1007/s10877-024-01181-y.

## Trial registration

NCT03144219, Registered 01 May 2017, https://www.clinicaltrials.gov/ct2/show/NCT03144219.

**Supplementary Information:**

The online version contains supplementary material available at 10.1007/s10877-024-01181-y.

## Highlights

Noninvasive methods as transcranial Doppler and a cranial pulsation sensor provided pathophysiological parameters which may be associated with poorer outcomes in acute brain injury.

P2/P1 ratio, eICP and eCPP demonstrated high negative predictive values for ruling out either intracranial hypertension or imminence of death.

The combination of these techniques was important to improve their predictive value, rather than using them separately.

The strong correlation of these parameters with outcomes but only moderate correlation with intracranial pressure values seems to reinforce the need for a physiological and dynamic reasoning when dealing with neurocritical patients and monitoring ICP.

**Supplementary Information:**

The online version contains supplementary material available at 10.1007/s10877-024-01181-y.

## Introduction

Acutely brain injured (ABI) patients are at high risk of death and poor functional outcomes worldwide [1, 2]. Beside primary injuries following kinetic forces in head trauma or spontaneous intracranial bleedings, further damage may occur because of the inflammatory and immune responses in the subsequent early period after a severe ABI [3, 4]. This combination of phenomena contribute to impairment in cerebrovascular properties, raising risks for the development of intracranial hypertension (IH), a life-threatening condition for the neurocritical patient [5–7].

These patients require multiple systemic and brain catheterization for their monitoring and treatment, turning medical practice in a carrier of additional risks. Therefore, noninvasive methods which may diminish medical practice burden are of great value. In this setting, transcranial Doppler (TCD) is an ultrasound-based technique able to perform multiple cerebrovascular diagnostics at bedside [8]. This technique has been extensively applied in neurocritical care for its potential to estimate intracranial pressure (ICP) and cerebral perfusion pressure (CPP) [9], with remarkable performance on discarding IH.

A distinct noninvasive approach tailored for evaluating loss of compliance on the intracranial compartment was recently released for application in medical standards (B4C system) [10]. This technique has the ability to register skull pulsations and present surrogate ICP waveforms (ICPW) in real-time, allowing physicians to observe the impact of their interventions at bedside [11].

The morphological changes in ICPW are markers of intracranial compliance (ICC) impairment [12–14]. Such changes are the targets of the B4C system [15], by means of the quotient between the second and first ICPW peaks (P2/P1). Since IH indicates a decrease in ICC, it can result in CPP reduction [16], indicating a potential role for noninvasive monitoring combination by means of TCD and B4C, although there is paucity of data in this regard. Hence, the present study aimed to assess the correlation between TCD features and P2/P1 either separately or in combination- with short-term outcomes in neurocritical patients (primary endpoint). Additionally, the correlation of these parameters with ICP was also tested (secondary endpoint). Hypothetically, the power of noninvasive neuromonitoring techniques correlations is enhanced when different techniques are combined [17–19].

## Methods

### Study design

We conducted a retrospective analysis of a prospective study database to investigate the prediction ability of multiple parameters for the detection of IH and patient outcome differentiation. This observational study was conducted in the Hospital das Clínicas intensive care units (São Paulo University, Brazil). The study protocol was approved by the local Ethics Committee and retrospectively registered at clinicaltrial.gov (NCT03144219). Informed consent was obtained from legally authorized representatives/next of kin of patients before inclusion. This study was performed according to the STARD (Supplemental material).

### Population

Severe traumatic brain injury (TBI) and subarachnoid hemorrhage (SAH) patients were included exclusively when an invasive ICP probe monitor was inserted, according to the guidelines of the Brain Trauma Foundation and after neurosurgical and neurointensivist teams judgement [20, 21]. Therefore, the study was executed after admission stabilization interventions, such as sedation and intubation, ICP monitor implantation and intracranial mass evacuations when needed. In this institution, in non-traumatic neurosurgical emergencies but at risk for brain herniation according to the team discretion, the implantation of ICP monitoring is proceeded.

Exclusion criteria was the evidence of fixed mydriatic/middle-sized pupils for more than 2 h after ventilatory and hemodynamic stabilization. Patients were assessed within the first 5 days after admission. Simultaneous recording of invasive arterial blood pressure (ABP), ICP, electrocardiogram, temperature and oxygen saturation were also obtained.

The process of weaning patients from mechanical ventilation was carried out by the local ICU team independently of this study, following established criteria for this purpose. These criteria included assessing the patient’s ability to safeguard their airway, ensuring the maintenance of airway patency, confirming the presence of a robust cough reflex, evaluating the Glasgow coma scale (GCS) score to be above 8, monitoring a PaO2/FiO2 ratio exceeding 150 and considering the necessity for FiO2 levels greater than 0.40 or a PEEP (positive end-expiratory pressure) requirement below 10. These stringent criteria were applied to ensure the safe and appropriate weaning of patients from mechanical ventilation.

Three short-term outcome (STO) groups were separated based on to Seattle International Brain Injury Consensus Conference therapy intensity level (TIL) recommendation (Table 1) [22]. Group 1 (STO 1) was composed by no specific ICP directed therapy patients, whilst group 2 (STO 2) by ICP directed TIL 1–3. Finally, group 3 (STO 3) was composed by in-ICU early deaths. These outcomes were registered at day 7 after last monitoring session; therefore, the outcomes of interest were registered in the second week of hospitalization, in order to allow potential associations of changes in the variables provided by noninvasive neuromonitoring (TCD and B4C) and clinical results in a short-term period.

**Table 1:** Expert consensus for traumatic brain injury therapy intensity level (TIL) escalation. (Adapted from Hawryluk et al.: A management algorithm for patients with intracranial pressure monitoring: the Seattle International Severe Traumatic Brain Injury Consensus Conference)

TIL 0	No specific ICP-directed therapy. Basic ICU care: sedation, vasopressors, overall variables control and head positioning not based on ICP management.
TIL 1	Mild ICP-directed therapy: Deeper sedation, vasopressors, osmotics, hypocapnia and CSF drainage according to ICP/CPP.
TIL 2	Moderate ICP-directed therapy: More rigorous TIL 2 plus mild hypothermia ˜35^0^C.
TIL 3	Extreme ICP-directed therapy: Deeper hypocapnia, hypothermia, barbiturates, neurosurgery.

### Data sources and procedures

TCD (Doppler Box, DWL, Singen, Germany) recorded cerebral blood velocities (CBv) from middle cerebral arteries bilaterally through the temporal acoustic windows. In case of more than 20% CBv values difference between sides, the side with higher injury (according to the admission CT scan) was chosen for the statistical analysis. P2/P1 was collected using the brain4care (B4C) system (Brain4care, São Carlos, Brazil). The P2/P1 is a parameter derived from the surrogate B4C waveforms, which are waveforms correlated with ICP changes [15], obtained after the discovery of the principle of skull microexpansion [23, 24]. ICP was measured using the Neurovent monitoring system via optic-fiber transducer (Raumedic, Munchberg, Germany); mean ICP values were registered for every monitoring session.

Patients were under sedation and mechanical ventilation. The monitoring sessions lasted about 10–15 min and all data (from ICP, TCD and B4C) were acquired simultaneously and electronically at a sampling frequency of 250 Hz (from DWL software for TCD and Phillips Mx800 monitor for ICP and B4C). Therefore, there was no time interval between the records of each technique.

**Fig. 1:**
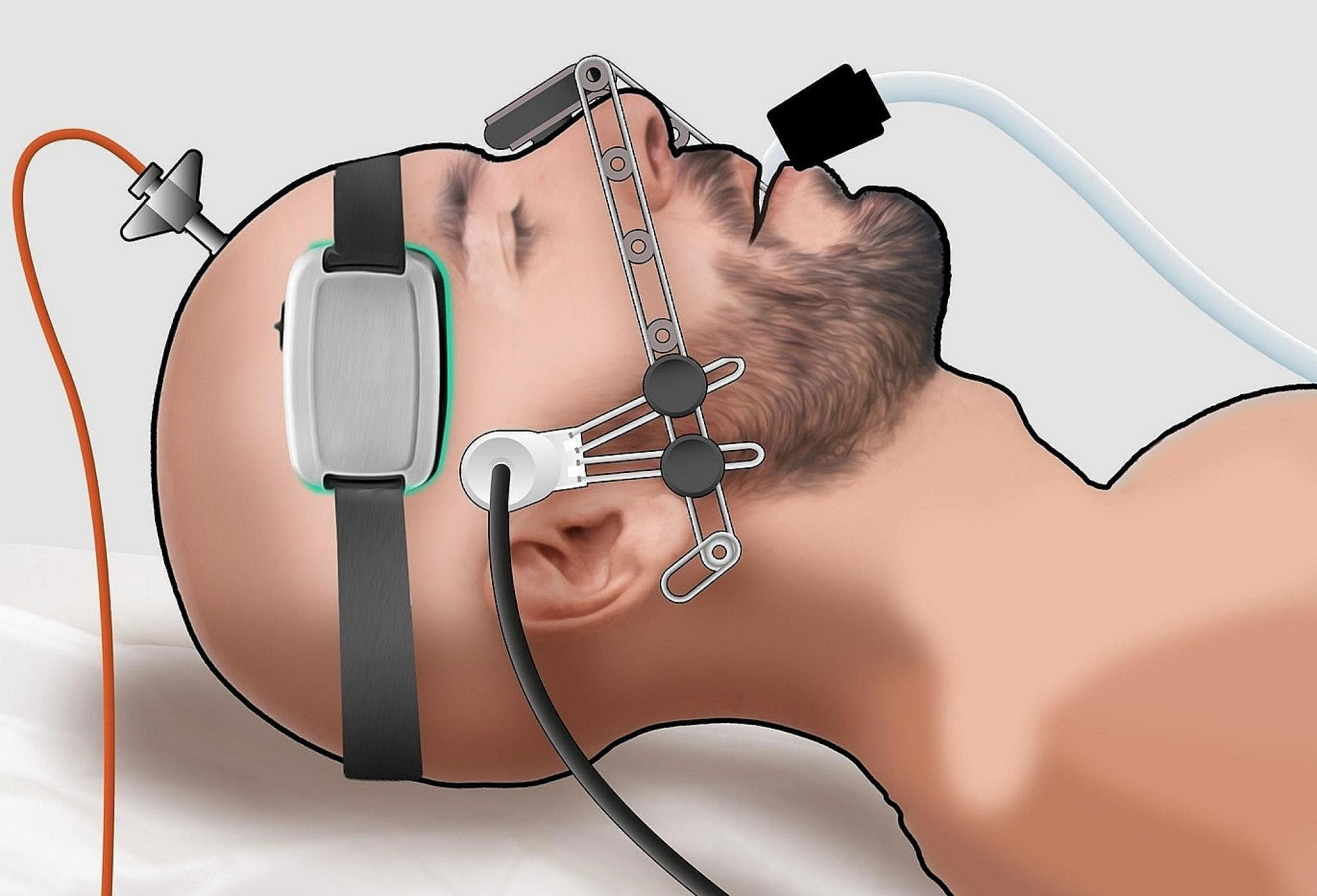
Schematic drawing of data collection setting. Intracranial pressure (ICP), B4C sensor and transcranial Doppler (TCD) data registered concomitantly

During these sessions, strict monitoring by the responsible investigator (SB) was maintained to prevent any displacement of the B4C and TCD sensors, which could potentially compromise the quality of the signals. All recordings were performed independently of therapeutic interventions, as well as therapy was not influenced by the parameters observed in this study (Fig. 1). The present database was built primarily to assess physiological properties of ICP changes over CH [16, 25]. Multiple parameters were collected but only a maximum of two sessions per patient in the first five days after hospitalization. Therefore, correlation with outcomes was limited to the TIL of this sample in the second week, precluding causality correlations. The study was limited exclusively to search for associations between a possibility of CH and ICC impairments to influence on STO.

### Data preprocessing

Prior to the analysis, the dataset underwent preprocessing steps to ensure data quality and compatibility, filtering to reduce signal-to-noise ratio and artifacts removal. The parameters of interest were, from TCD: systolic (sCBv), diastolic (dCBv) and mean CBv (mCBv), pulsatility index (PI, formula: PI = sCBv-dCBv/mCBv), estimated eCPP (eCPP, formula: eCPP = meanABPxdCBv/mCBv + 14) and estimated eICP (eICP, formula: eICP = MAP-eCPP) [19]. From B4C, the P2/P1, which represents the marker of buffering reserve exhaustion and the move towards IH [26]. Both techniques (B4C and TCD) parameters have demonstrated significant correlations with ICP in previous studies [15, 19, 27]. These variables were all recorded concomitantly with ICP and their mean values for the duration of each monitoring session were calculated automatically for statistical analysis. IH was defined in this study as ICP > 20 mmHg.

### Data analysis

Data analysis was performed in two ways. First, each proposed method was evaluated separately as to their abilities to predict the presence or absence of IH and differentiating favorable outcomes and death (STO 1 and 3). Secondly, P2/P1 and each respective parameter derived from TCD were combined. The following steps were performed for this secondary analysis:

1. Splitting the Data: the dataset was randomly divided into a training set and a testing/validation set using a 50:50 ratio. The training set (*N* = 49 measurements) was used for model development, while the testing/validation set (*N* = 49 measurements) was used for model evaluation. Supplemental tables provided full description of these cohorts.
2. Logistic Regression Model: a logistic regression model was constructed using the training set. The response variables were “intracranial hypertension” and “outcomes” (STO levels 1 and 3) which were encoded as binary variables (0 for absence, 1 for presence) in separate columns in the dataset. The predictor variables included the selected parameters. The logistic regression model was fitted using the `glm()` function from the R statistical software, with the family set to “binomial”.
3. Model Evaluation: the fitted logistic regression model was then used to generate predictions on the testing/validation set. Predicted probabilities were obtained using the `predict()` function. To assess the performance of the model, a receiver operating characteristic (ROC) curve analysis was conducted. The ROC curve was plotted using the `pROC` package in R [28], and the area under the ROC curve (AUC-ROC) was calculated as a measure of the model’s predictive ability.

### Statistical analysis

Continuous and categorical variables were summarized differently: absolute (n) and relative frequencies (percentage) were used for qualitative variables, while mean ± standard deviation and medians with minimum and maximum values were employed for quantitative variables. The correlation between the noninvasive methods and the absolute value of intracranial pressure (ICP) was evaluated using Spearman’s correlation coefficient. To assess the effectiveness on distinguishing between favorable outcome and non-survivors (STO 1 and 3, respectively), the area under the curve (AUC) of the receiver operating characteristic (ROC) curve was calculated for each method. Additionally, an analysis was conducted to determine optimal cutoff values for predicting ICP > 20 mmHg using the Youden index. These values represent the optimal balance between sensitivity and specificity for a given threshold. An AUC exceeding 0.7 indicates reasonable predictive ability, while an AUC surpassing 0.8 signifies strong predictive ability [29]. Statistical comparisons between ROC curves were performed using DeLong’s test for two correlated ROC curves (R package pROC [28]). To jointly analyze the relationships of these methods with outcomes, binary logistic regression models were employed, utilizing generalized estimated equations. The findings were expressed as odds ratios (ORs) along with their respective 95% confidence intervals and *p*-values. The dependent variables were the outcomes, whereas the independent variables were the noninvasive methods. Additionally, an analysis of variance (ANOVA) was executed to explore potential associations between the assessed variables and the different STO levels within the patient cohort. All statistical analyses were carried out using RStudio (R version 4.3.1), with statistical significance determined by a *p*-value threshold of 0.05.

## Results

152 patients were consecutively included but only 98 of these had both TCD and B4C recorded for analysis. Patients were middle-aged (41 ± 21 y.o.) predominantly male (65%) and hospitalized because of TBI (70%) as shown in Table 2. Patients who had suffered SAH (*n* = 29) were all because intracranial aneurysm rupture. At enrollment, 18 (62%) of these had been submitted to coiling whereas the remaining 11 to clipping microneurosurgery. 30 patients (30%) presented IH during recording sessions, 19 instances of IH (in 19 respective patients) were detected for the analysis contemplating individual methods (overall population cohort), while 11 instances (in 11 respective patients) were identified in the subset of 50 measurements from the testing dataset for the analysis contemplating combined methods (TCD + B4C). The analysis was also conducted at different STO levels. At levels 1 and 3, 37 measurements were considered from the original dataset, with 17 measurements showing STO 3 for the individual methods analysis. Additionally, from the testing dataset (*N* = 50), 14 measurements were identified at STO levels 1 and 3, and out of these, 8 measurements exhibited level 3 for the combined methods analysis. No significant differences regarding admission characteristics in the population assessed were found.

**Table 2:** Baseline characteristics of the patient cohort and mean (SD) values of the studied parameters

	All (*n* = 98)	STO 1 (*n* = 20)	STO 2 (*n* = 62)	STO 3 (*n* = 16)
Age, years	41.90 (21.11)	38.55 (18.82)	40.59 (21.23)	50.65 (22.08)
Sex, n males (%)	64 (65)	14 (70)	41 (66)	9 (56)
**Parameters**				
mICP, mmHg	14.70 (7.60)	14.10 (5.60)	12.97 (5.24)	21.59 (12.37)^c^
mICP ≥ 20 mmHg, n (%)	19 (19)	4 (20)	6 (10)	9 (56)
P2/P1	1.10 (0.27)	1.05 (0.24)	1.08 (0.27)	1.27 (0.23)^b, c^
P2/P1 ≥ 1.2, n (%)	40 (41)	8 (40)	21 (34)	11 (69)
MAP, mmHg	89.60 (11.40)	88.55 (10.48)	90.17 (11.77)	88.65 (11.55)
CBv, cm/s	79.10 (28.20)	78.38 (26.63)	79.83 (28.90)	77.32 (29.23)
PI	0.90 (0.25)	0.89 (0.21)	0.87 (0.20)	1.04 (0.37)
eICP, mmHg	18.80 (11)	19.56 (9.65)	16.69 (7.90)	26.02 (18.11)
eCPP, mmHg	70.50 (13.70)	71.76 (12.64)	72.47 (12.74)	61.54 (15.67)^c^
SO_2_ (%)	98 (3)	98.30 (2.43)	98.03 (3.32)	97.41 (2.37)
PaCO_2_, mmHg	37 (5.49)	37.30 (7.03)	36.6 (5.29)	36.15 (3.67)
Hemoglobin, mg/dL	9.56 (1.65)	9.60 (1.73)	9.50 (1.53)	9.72 (2.07)
Temperature, ºC	36.12 (0.93)	36.78 (0.81)	36.06 (0.90)^a^	35.56 (0.74)
**Pathology**				
TBI	69 (70)	18	45	6
Marshall score II	7	5	2	0
Marshall score III	50	13	36	1
Marshall score IV	12	0	7	5
SAH	29 (30)	2	17	10
mFS 3	8 (27)	2	3	3
mFS 4	21 (77)	0	14	7
**Neurosurgery**				
No	27	5	14	7
Craniotomy	42	9	20	4
Craniectomy	31	6	28	5
**Comorbidities**				
None	65	12	44	9
Metabolic Syndrome	21	4	14	3
Others	12	4	4	4
SAPS-3 Score	58.84 (12.22)	54 (10.40)	59.63 (12.23)	61.65 (13.29)
Admission GCS	5.73 (4.40)	5.75 (4.35)	5.92 (4.58)	5 (3.95)

a: indicates significant statistical difference between STO groups 1 and 2

b: indicates significant statistical difference between STO groups 1 and 3

c: indicates significant statistical difference between STO groups 2 and 3

CBv: cerebral blood velocities, eCPP: estimated cerebral perfusion pressure, mICP: mean intracranial pressure, eICP: estimated intracranial pressure, GCS: Glasgow coma score, MAP: mean arterial pressure, mFS: modified Fisher score, PaCO_2_: carbon dioxide pressure, PI: pulsatility index, SAH: subarachnoid hemorrhage, SAPS3: simplified acute physiologic scale, SO_2_: oxygen saturation, STO: short-term outcome, TBI: traumatic brain injury

### TCD and B4C results

**Fig. 2:**
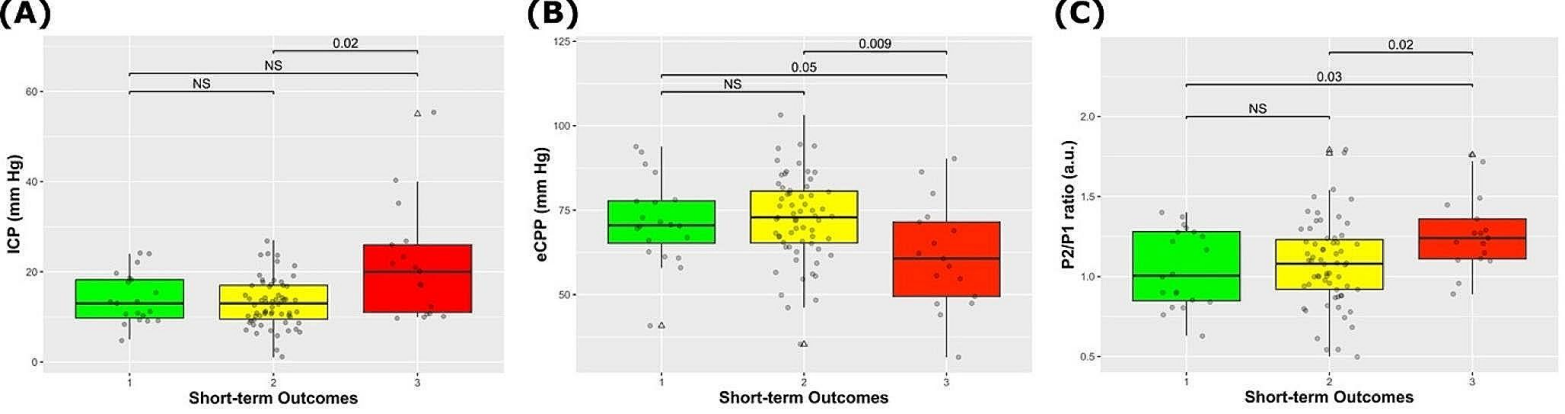
Most significant variables associated with short-term outcomes. eCPP and P2/P1 were significantly different between survivors and non survivors. eCPP: estimated cerebral perfusion pressure

Detailed results of the receiver operating characteristic (ROC) analysis, encompassing metrics such as AUC, sensitivity, specificity, accuracy, positive predictive value (PPV), negative predictive value (NPV), are outlined in Table 3. P2/P1 and eCPP were the parameters significantly different between both survivor groups (STO 1 and 2) and the STO 3 group (Fig. 2). For mortality prediction, the P2/P1 exhibited an AUC of 0.70 (95% CI 0.53–0.87), alongside a strong NPV of 100%.

**Table 3:** Results of receiver operating characteristic analysis for mortality (*N* = 37 measurement points of the original dataset for single methods; *N* = 14 measurement points of the testing dataset for combined methods). mCBv: mean cerebral blood velocities, eCPP: estimated cerebral perfusion pressure, eICP: estimated intracranial pressure, ICP: intracranial pressure, PI: pulsatility index, P2/P1: quotient from second and first brain4care waves peaks

Parameters	AUC (95% CI)	Sensitivity (%)	Specificity (%)	Accuracy (%)	PPV (%)	NPV (%)
ICP	0.69 (0.52–0.87)	100	5	49	47	100
P2/P1	**0.70 (0.53–0.87)**	100	5	49	47	100
mCBv	0.47 (0.28–0.67)	100	5	49	47	100
PI	0.62 (0.42–0.81)	94	0	43	44	0
eICP	0.59 (0.40–0.78)	100	5	49	47	100
eCPP	**0.70 (0.52–0.88)**	100	5	49	47	100
P2/P1 + PI	**0.83**	100	17	64	61	100
P2/P1 + mCBv	**0.81**	100	17	64	61	100
P2/P1 + eICP	**0.71**	100	17	64	61	100
P2/P1 + eCPP	**0.85**	100	17	64	61	100

**Table 4:** Results of receiver operating characteristic analysis for ICP > 20 mmHg. All parameters except for PI demonstrated to have excellent NPV. AUCs became accepetable when P2/P1 was combined with either PI or eICP. mCBv: mean cerebral blood velocities, eCPP: estimated cerebral perfusion pressure, eICP: estimated intracranial pressure, PI: pulsatility index, P2/P1: quotient from second and first brain4care waves peaks, PPV: positive predictive value, NPV: negative predictive value

Parameters	AUC (95% CI)	IH cutoff	Sensitivity (%)	Specificity (%)	Accuracy (%)	PPV (%)	NPV (%)
P2/P1	0.66 (0.53–0.78)	1.13	100	1	20	19	100
PI	0.66 (0.51–0.82)	0.96	95	1	19	18	19
mCBv	0.67 (0.41–0.69)	92.65	100	1	20	19	100
eICP	0.67 (0.50–0.83)	25.25	100	1	20	19	100
eCPP	0.67 (0.51–0.82)	64.09	100	1	20	19	100
P2/P1 + PI	**0.70**	-	100	3	24	22	100
P2/P1 + mCBv	0.67	-	100	3	24	22	100
P2/P1 + eICP	**0.72**	-	100	3	24	22	100
P2/P1 + eCPP	0.67	-	100	3	24	22	100

### Combined methods

In the prediction analysis of STO 3, the top-performing methods included “P2/P1 + eCPP,” “P2/P1 + PI,” and “P2/P1 + CBv,” achieving AUCs of 0.85, 0.83, and 0.81, respectively. All these methods showcased robust NPVs of 100% and moderate PPVs of 61% (Table 3). Table 5 presents a comprehensive summary of the outcomes derived from logistic regression models based on combined noninvasive methods. To distinguish IH, the methods “P2/P1 + PI” and “P2/P1 + eICP” emerged as the best performers, showing AUCs of 0.70 and 0.72, respectively. Moreover, they demonstrated NPVs of 100% (Fig. 2) (Table 4). Despite marginally enhanced AUCs for IH assessment, these combined methods did not exhibit statistical significance when compared to the individual methods (P2/P1, PI, and eICP).

**Table 5:** Summary of the logistic regression results for the combined noninvasive methods

Model	Predictor	Odds Ratio	95% CI Lower	95% CI Upper	*p*-value
P2/P1 + PI	Intercept	0.0006	0.00003	0.12	0.006
	P2/P1	8.81	0.38	203.76	0.17
	PI	26	1.34	503.58	0.03
P2/P1 + mCBv	Intercept	0.01	0.0002	0.61	0.03
	P2/P1	6.19	0.33	116	0.22
	CBv	1.01	0.99	1.03	0.43
P2/P1 + eICP	Intercept	0.01	0.0002	0.50	0.02
	P2/P1	4.77	0.23	99.15	0.31
	eICP	1.05	0.97	1.15	0.24
P2/P1 + eCPP	Intercept	1.90	0.01	320.55	0.81
	P2/P1	10.08	0.35	286.81	0.18
	eCPP	0.92	0.86	0.99	0.03

mCBv: mean cerebral blood velocities, eCPP: estimated cerebral perfusion pressure, eICP: estimated intracranial pressure, PI: pulsatility index, P2/P1: quotient from second and first brain4care waves peaks

### Relationship with short-term outcomes

**Fig. 3:**
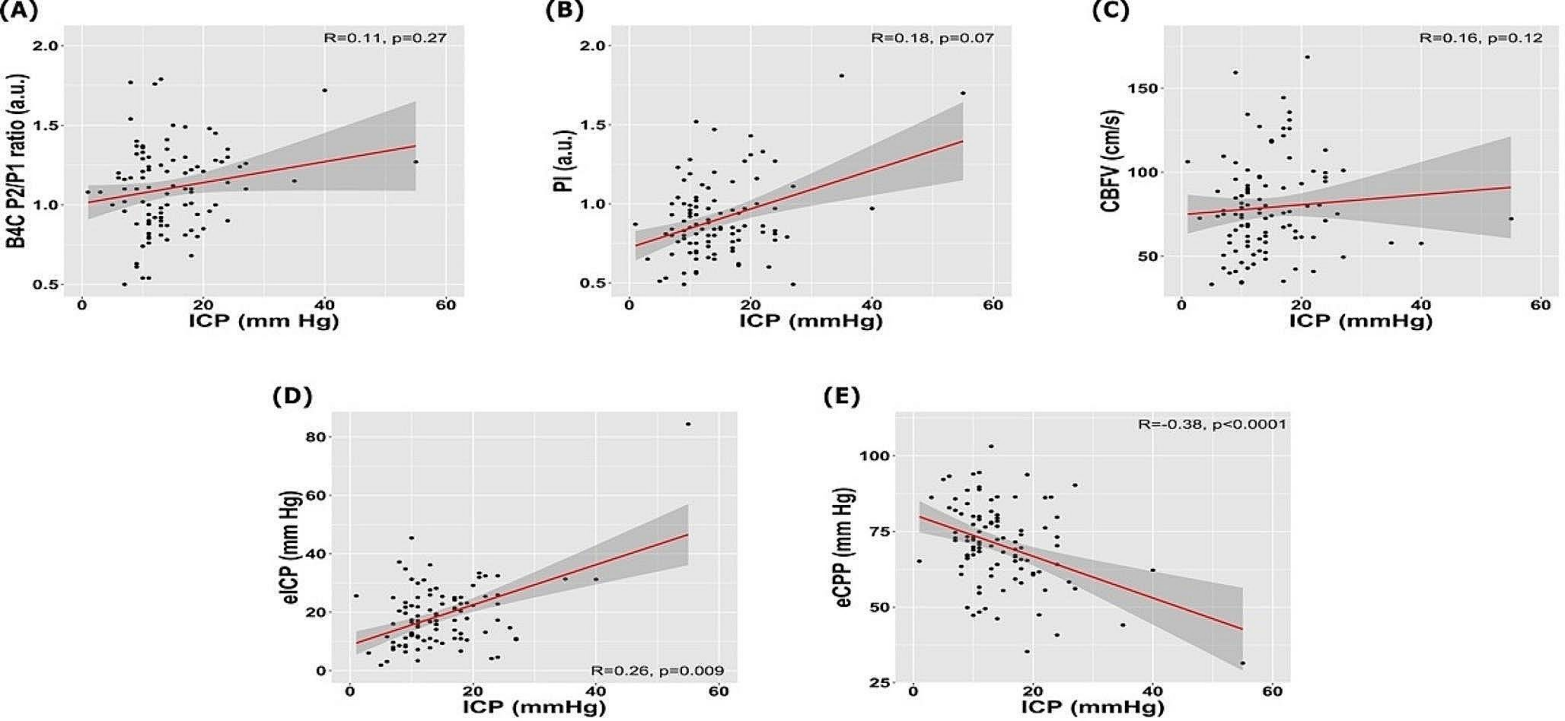
Linear correlations with intracranial pressure (ICP) for the parameters assessed. CBFV: cerebrovascular velocities, eCPP: estimated cerebral perfusion pressure, eICP: estimated ICP, P2/P1: quotient between second and first ICP peaks, PI: pulsatility index

16 patients died (STO 3) and 82 survived (STO levels 1 and 2). 12 deaths (75%) due to brain death and the remaining 4 among patients older than 65 years with previous comorbities. Twenty patients had most favorable outcomes (STO 1). Figure 3 depicts the noninvasive methods that presented at least one association with patient outcome.

The methods that presented significant difference between favorable outcome and non-survivors were eCPP and P2/P1 (Fig. 2). Among these, P2/P1 demonstrated the highest significance level (*p* = 0.03) to differentiate these outcomes. ICP was significantly higher in patients who died compared to those of group STO 2. STO groups 1 and 2 median ICP levels were under 20 mmHg, whereas the opposite succeeded for the mortality group.

## Discussion

In the present cohort of 98 acute brain injured patients, two noninvasive techniques were used to register biometrical parameters for the assessment of ICC and CH. CH and ICC impairments were indicators of poorer short-term outcomes. Furthermore, the severity of such disturbances observed in the early days after injury was a reliable prognostic factor, being ICP, P2/P1 and eCPP factors significantly associated with early death in the ICU. On the contrary, patients who exhibited early progression toward successful spontaneous breathing showed either mild or no CH and ICC impairment. These significant correlations with outcomes but moderate associations between CH and ICC with IH might reinforce the inaccuracy of such thresholds (as > 20 mmHg) to differentiate patients at stake of inadvertent secondary injuries.

The prognostic power by means of the P2/P1 was modest but with excellent NPV, indicating this parameter between 0.8 and 1.1 valuable to rule out ICC impairment. In the present study, a mean P2/P1 over 1.2 was found as indicator of unfavorable outcome, which is in concordance with previous studies [26, 30]. Regarding the several TCD parameters, eCPP was significantly lower in the group of patients who died, but overall, TCD parameters also disclosed strong NPVs and moderate PPVs. However, when taken in combination, TCD and B4C prognostication power was enhanced, as demonstrated by de Moraes et al. in a recently published study assessing patients with SAH [31]. These findings remark the prognostic role of noninvasive neuromonitoring, pointing to the possibility of targeting therapies on such information in future studies.

It has been reported that neurocritical patients exhibit an extended duration of mechanical ventilation and a heightened prevalence of extubation failure in comparison to the broader critically ill [32]. Notably, in studies specifically investigating patients with ABI, a noteworthy proportion of approximately 35% needed the implementation of tracheostomy procedures [32]. Furthermore, a systematic review including 7929 patients indicated that several parameters are considered for the decision on mechanical ventilation weaning, being age, level of consciousness, the inspiratory maximum pressure, the rapid shallow breathing index and overall disease severity scales the most used [33]. However, these parameters are assessed concomitantly with sedation weaning. It is important to highlight that none of these studies assessed brain dynamic conditions as CH and ICC as markers of brain health to support mechanical ventilation withdrawal, which according to the findings of the present study may be advocated as ancillary information to assess in such process.

Robba et al. previously observed a prediction power improvement of combining TCD, pupillometry and optic nerve sheath ultrasound, rather than using such techniques separately [17, 34]. Godoy et al. proposed a model for the combination of these same techniques but including also ICP pulse morphology with the purpose of understanding further the intracranial compartment syndrome [18]. Brasil et al. assessing 72 ABI patients observed the potential refinement of ICP invasive monitoring coupled with B4C waveforms on outcome prediction, advocating not only for the potential benefit on adding noninvasive techniques even when an ICP catheter is implanted [26].

TCD was concentrated in the past on the PI as maker of raised ICP [35]. Notwithstanding, many studies brought controversial results for the PI in this regard [36–38], concluding this parameter to be more an indicator of reduced CPP (when PI is raised > 1.4) than elevated ICP [39]. Our study is in accordance with this latter point of view. The sample of this study was composed predominantly by severe TBI patients, which in early days after injury often present posttraumatic hyperemia [3]. We observed elevated mean CBv (> 70 cm/s) [40] and low PI (˜0.9) in all groups, what reinforce this point. Therefore, calculating eICP and eCPP is advised when using TCD to evaluate neurocritical patients in the early days after injury. Additionally, as demonstrated, being B4C available, its use altogether with TCD will probably enhance diagnostic power.

In acknowledging the limitations of our study, we recognize the absence of a longer-period cohort as a primary constraint, which could have facilitated more robust associations between parameters and outcomes. It is pertinent to note, however, that there is a scarcity of literature focusing on short-term outcomes within this specific patient group. Furthermore, the study acknowledges the potential occurrence of some IH surges that may not have been captured. Owing to the limited number of datapoints recorded, the associations assessed do not establish causality between parameters and outcomes. It is essential to acknowledge that various other variables, not measured in this study, play fundamental roles in determining outcomes. Nevertheless, the absence of significant clinical differences between groups at least raises the possibility that the present analysis holds true relevance. Furthermore, it is essential to acknowledge the relatively smaller sample size and the limited number of cases with ICP exceeding 20 mm Hg. The constrained sample size presents inherent challenges, and while logistic regression analysis has been employed with meticulous consideration of its limitations, the findings must be interpreted with caution. The preponderance of observations with ICP < 20 mmHg and the modest correlations between certain variables are acknowledged limitations. The low specificity and positive predictive values, particularly the 1% specificity for ICP > 20 mm Hg presented by P2/P1, suggests potential challenges in its role as a standalone diagnostic parameter.

## Conclusions

Noninvasive cerebral hemodynamics and intracranial compliance assessments seem to be associated with early clinical outcomes after acute brain injuries. Such potential was enhanced when considering a combination of techniques, supporting the use of multimodal monitoring in neurocritical care. The results of this exploratory investigation advocate for prospective studies and definition on strategies for ABI management using these tools in addition to ICP monitoring.

## Electronic supplementary material

Below is the link to the electronic supplementary material.

Supplementary Material 1

## Data Availability

No datasets were generated or analysed during the current study.

## Abbreviations

ABI: Acute brain injury
ABP: Arterial blood pressure
AUC: Area under the curve
B4C: Brain4care
CPP: Cerebral perfusion pressure
ICC: Intracranial compliance
ICP: Intracranial pressure
IH: Intracranial hypertension
NPV: Negative predictive value
SAH: Subarachnoid hemorrhage
STO: Short-term outcomes
TBI: Traumatic brain injury
TCD: Transcranial Doppler
